# Trachoma and women: latrines in Ethiopia and surgery in Southern Sudan

**Published:** 2009-06

**Authors:** Paul M Emerson, Lisa Rotondo

**Affiliations:** Director, Trachoma Control Programme, The Carter Center, 1 Copenhill, Atlanta, GA 30307.; Associate Director, International Trachoma Initiative, Task Force for Global Health, 325 Swanton Way, Decatur, GA 30030.

**Figure:**
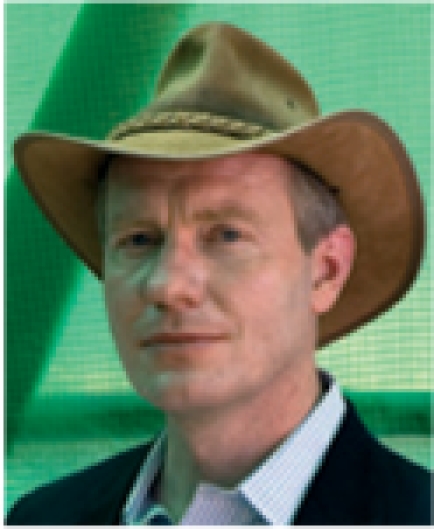


**Figure:**
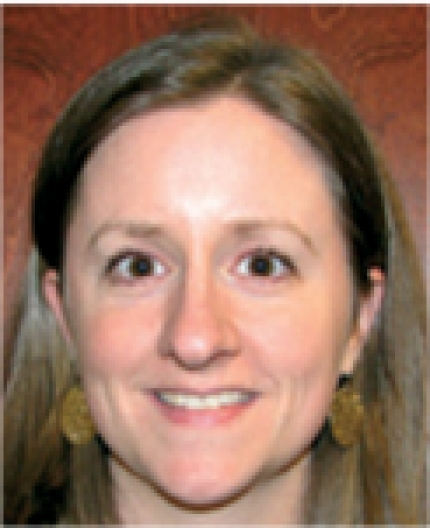


Trachoma is an infectious disease of the eye caused by the bacterium *Chlamydia trachomatis*. Bacteria can spread via an infected person's hands or clothing and may be carried by flies that have come into contact with discharge from the eyes or nose of an infected person.

Infants and children below school age are more likely to be infected. Since trachoma is transmitted through close personal contact, it often infects children in entire communities.

Although children are more susceptible to infection, the painful and often blinding complication of trachoma – trachomatous trichiasis – usually does not appear until adulthood. Trachomatous trichiasis is the result of repeated infections by *Chlamydia trachomatis* which cause scarring of the inner surface of the upper eyelid; this eventually causes the eyelashes to turn inward and scratch the cornea, causing corneal opacity and pain. Unless this process is halted early enough, a person with trachomatous trichiasis will become blind.

WHO recommends the SAFE strategy to control trachoma:

- **S**urgery to reverse the in-turning of the eyelid and eyelashes, relieving pain and sometimes preventing blindness.
- **A**ntibiotics (azithromycin) to treat active trachoma and decrease the burden of infection in a community.
- **F**acial cleanliness or the incorporation of good hygiene practices, including hand washing.
- **E**nvironmental improvements to reduce the transmission of the disease, such as latrines (to reduce flies) and water for face and hand washing.

Trachomatous trichiasis affects nearly twice as many women as men. The SAFE strategy should be targeted at all people in areas where trachoma is endemic, but specifically at women and children in order to address this inequality.

Although there may be an underlying biological reason that more women are affected by trachoma and trichiasis, the role of women as childcare providers is a likely cause. In most countries where trachoma is endemic, girls grow up in environments where one of their primary activities is taking care of their younger family members and siblings. This continues into adulthood, with women carrying the main responsibility of caring for children. During their lifetime, women therefore spend more time in direct contact with children who may be infected.

**Figure:**
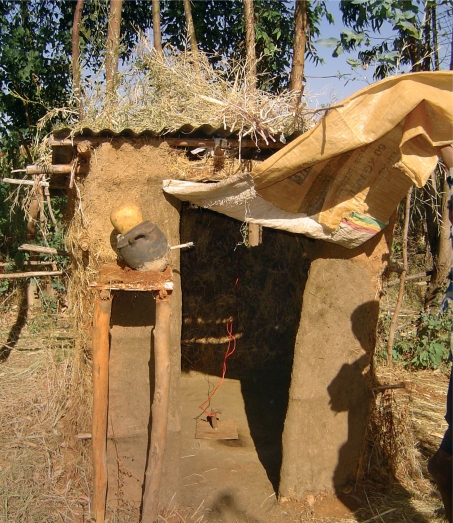
A demonstration latrine in a school is built of mud, grass, wooden sticks, and various household items. ETHIOPIA

Lisa Rotondo

Ethiopia and Southern Sudan are two locations with an exceedingly high burden of trachoma. Projects focusing on environmental improvement (in Ethiopia) and increasing access to surgery (in Southern Sudan) have made significant progress towards reducing the impact of the disease on women. These examples show how trachoma programmes can address the particular needs of women while designing interventions aimed at eliminating blinding trachoma in the community as a whole.

**Figure:**
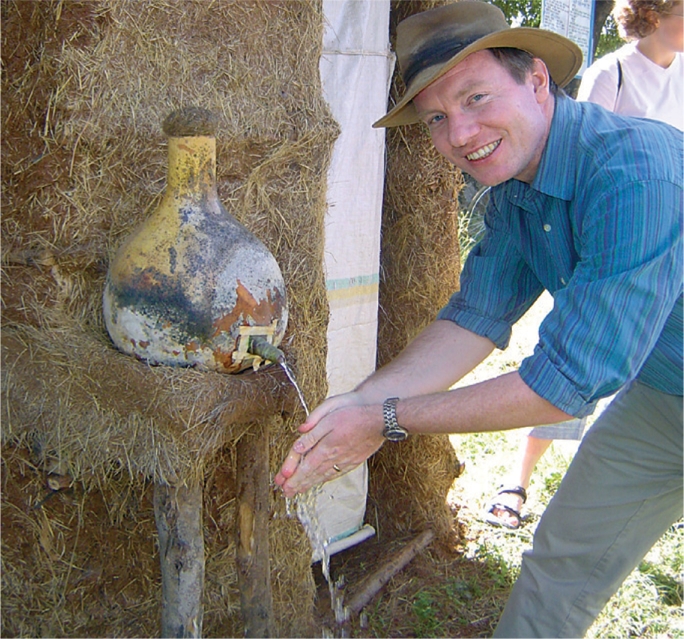
Paul Emerson tests out a household latrine's hand washing station. ETHIOPIA

Lisa Rotondo

## 1  Latrines in Ethiopia

Traditionally, community members in the Amhara region of Ethiopia go to the woods or fields to defecate. Women in particular are discouraged from defecating or urinating where they could be seen during the day and usually have to wait until the night to relieve themselves.

As part of the implementation of the full SAFE strategy, the government health office in the Amhara region worked with its partners to encourage communities to construct household pit latrines. Demonstration latrines were built in district health centres and primary schools to illustrate the ease with which a latrine can be constructed using materials readily available in the community. This project was highly successful: communities built more than ten times the expected number of latrines.

After investigating the reasons for this unexpected success, the programme discovered that it was women who championed latrine construction in their homes and communities. They encouraged their husbands and family members to work together to clear land near their homes, to dig pits, to gather local resources, and to build structures to enclose the pits. Widows and the disabled were helped by able-bodied friends and relatives. Latrine structures consisted of whatever materials a family had on hand: sticks, mud, tree branches, gourds, plastic sheeting, and so on. Many families constructed a hand washing station, also made of local materials, next to the latrine to encourage proper hygiene.

Using a household latrine reduced the population of flies transmitting the bacteria that cause trachoma. The privacy provided by the latrines also allowed women the freedom to relieve themselves when they needed to during the day and improved their safety as they no longer had to go far from their homes after dark. This helped to address some of the inequalities women faced in their homes and communities.

By actively leading the latrine construction movement, women have not only helped themselves, but have served their communities in the fight against trachoma. The trachoma programme has since used the knowledge gained from this experience to continue to address gender issues in trachoma and target latrine promotion programmes at women.

**Figure:**
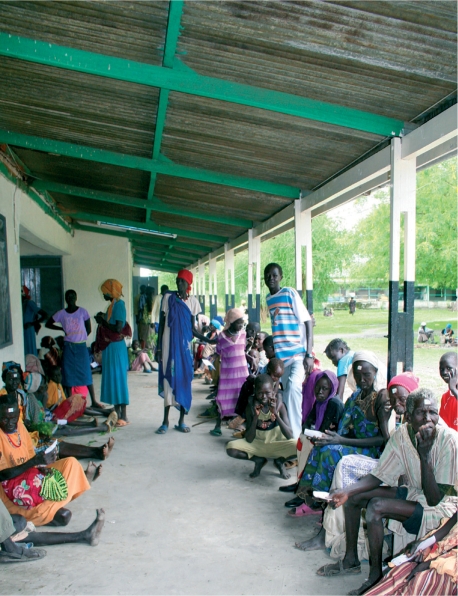
Community members wait for trichiasis surgery services. SOUTHERN SUDAN

The Carter Center Southern Sudan

## 2  Improving access to surgery in Southern Sudan

The prevalence of trachoma in Southern Sudan is very high. As in many trachoma-endemic areas, women are at greatest risk of being blinded by trachomatous trichiasis.

Southern Sudan is a very poor, rural region, where most people are subsistence farmers. In such an environment, a woman with advanced trichiasis becomes a burden to her family as she is unable to tend to her household chores, care for children, or contribute to the family economy. However, it is precisely these responsibilities that can make it difficult for a woman to leave her home and travel to a place where eyelid surgery is offered before she becomes irreversibly blind.

If families are not aware that a woman's trichiasis can be treated, or if it is not possible for such a woman to go where she can receive treatment, there is a risk that she will be abandoned by her family. It is therefore important that programmes do everything within their power to ensure that women and their families are aware of the services available and are able to make use of them.

The Carter Center is supporting a programme by the Southern Sudan Ministry of Health to improve access to surgery. In Jonglei State, the project has constructed a primary eye care clinic and trained local nurses in trichiasis surgery. After certification, the new trichiasis surgeons were provided with surgical instrument kits and consumables.

The local community decided where the clinic should be located; they chose a place that is easily accessible year-round and that is known to many communities.

Surgery is offered free of charge, reducing the barrier of cost, and is provided during routine services at the clinic and during outreach campaigns.

The clinic has been a successful mechanism for reaching the large population affected with trachomatous trichiasis in Southern Sudan and has become well known in the state, with patients from beyond the original target area walking for days to access its services.

**What can we do?**

- **Help to build latrines.** Women with trichiasis often end up without husbands. They are often unable to build latrines themselves and yet they and their children need them most. Identify such women and ensure that communities and professionals assist them with latrine building.
- **Identify the best way to reach women.** Depending on the community's needs, health professionals may need to counsel women and women's groups about the importance of treatment. They may also need to talk to the men and elders of the community about the importance of treatment for women and how this can benefit families and the community. There are women's groups in many communities; encourage them to coordinate women who can travel to clinics together.
- **Choose the best location for clinics.** Try and place surgeons in areas of high prevalence. Consider ease of access for patients and for delivery of supplies, as well as the availability of basic facilities such as clean water and latrines.
- **Bring surgery to the community.** Outreach campaigns can be organised in schools, religious centres, and other buildings and women can be specifically targeted. Campaigns must be very carefully planned and should include extensive information campaigns (see below), adequate quantities of consumable and non-consumable equipment, and participation by surgeons who are willing and able to operate on many patients each day.
- **Inform communities about services.** Tell communities repeatedly where their nearest surgical service is, the times when surgeons operate, and the exact dates of surgical outreach campaigns. Use loudspeakers in markets and ask for help from community leaders, religious centres such as churches and mosques, and schools (schools can encourage children to inform their mothers about surgical services). Attendance will improve if communities are also told that surgery is free.
- **Provide high quality surgery.** Several studies have shown that the risk that trichiasis will recur varies from one surgeon to the next. It is therefore essential that surgeons are well trained and certified and regularly receive refresher training. This is the best way to avoid recurrence and other complications. Patients, and particularly women, may not attend clinic again, so it is essential that they receive a high quality operation when they do come. The importance of postoperative eye care should be explained to patients and their families so as to reduce the risk of infection.

